# Digital integration of narrative medicine and patient-reported outcome measures to improve understanding of quality of life in metastatic breast cancer: the PERGIQUAL study

**DOI:** 10.1093/oncolo/oyaf367

**Published:** 2025-11-05

**Authors:** Alessandra Fabi, Maria Cecilia Cercato, Alessandro Rossi, Patrizia Bianchini, Marika D’Oria, Antonella Palazzo, Luisa Carbognin, Valentina Frescura, Giovanni Scambia, Diana Giannarelli, Massimo Di Maio, Cristina Cenci

**Affiliations:** Precision Medicine in Senology Unit, Department of Woman and Child Health and Public Health, Scientific Directorate, Fondazione Policlinico Universitario A. Gemelli IRCCS, Rome 00168, Italy; Digital Library ‘R. Maceratini’, IRCCS Regina Elena National Cancer Institute, Rome 00144, Italy; Precision Medicine in Senology Unit, Department of Woman and Child Health and Public Health, Scientific Directorate, Fondazione Policlinico Universitario A. Gemelli IRCCS, Rome 00168, Italy; Digital Narrative Medicine (DNM Srl), Rome 00161, Italy; Independent Researcher, Rome, Italy; Medical Oncology, Università Cattolica del Sacro Cuore, Rome, Italy; Comprehensive Cancer Center, Fondazione Policlinico Universitario A. Gemelli IRCCS, Rome, Italy; Precision Medicine in Senology Unit, Department of Woman and Child Health and Public Health, Scientific Directorate, Fondazione Policlinico Universitario A. Gemelli IRCCS, Rome 00168, Italy; Medical Oncology, Università Cattolica del Sacro Cuore, Rome, Italy; Division of Gynecologic Oncology, Department of Woman and Child and Public Health, Fondazione Policlinico Universitario Agostino Gemelli IRCCS, Rome 00168, Italy; Biostatistic, Fondazione Policlinico Universitario A. Gemelli IRCCS, Rome 00168, Italy; Department of Oncology, University of Turin, Molinette Hospital, Turin 10126, Italy; Digital Narrative Medicine (DNM Srl), Rome 00161, Italy

**Keywords:** narrative medicine, patient-reported outcome measures, quality of life, breast cancer

## Abstract

**Purpose:**

This observational, prospective study aimed to evaluate the feasibility and usefulness of a person-based care (PbC) digital pathway integrating narrative medicine (NM) and electronic patient-reported outcomes measures (e-PROMs) for patients with metastatic breast cancer; the secondary objective was to assess its perceived impact on quality of life (QoL).

**Patients and methods:**

The study was conducted in Italy from May 2023 to February 2024. Enrolled patients (≥18 years old, life expectancy ≥24 weeks) completed a digital diary with narrative prompts and the EORTC QLQ-C30/+Br23 questionnaire at enrollment and before each treatment cycle. Oncologists reviewed narratives and patient-reported outcomes (PROs), adjusting care accordingly. Feasibility, usefulness, and perceived impact on QoL were assessed through final questionnaires for patients and oncologists. Feasibility was also evaluated via compliance and usefulness through thematic and comparative analysis of narratives and PROs, assessing integration, concordance, and discordance.

**Results:**

Twenty-nine patients and 6 oncologists participated. Study compliance was high; 21 patients and all oncologists completed the final questionnaires. Patients evaluated the digital diary as usable and effective in communication, while oncologists valued its compatibility with clinical workflow and ability to gather details not captured by ePROMs. Concordance and discordance between narratives and PROs were low (4.8% vs. 0.5%), but they provided complementary information (30.5% vs. 64.2%). PROs were more integrative in “problem” items (78.4% vs. 15.5%), whereas narratives were more informative on “resource” items (79% vs. 18.3%). Ten patients reported improved perceived QoL through the PbC pathway, while 6 found it potentially beneficial. Oncologists recognized its role in personalizing care.

**Conclusion:**

Integrating NM and ePROMs in the PbC pathway proved feasible and useful, enhancing the perceived impact on QoL and fostering more personalized and empathetic care.

Implications for Practice Patient-reported outcomes and narratives provided complementary information on disease and treatment trends; oncologists recognized the value of this integration in tailoring care in metastatic breast cancer.

## Introduction

Breast cancer (BC) is the most prevalent neoplasm and the fifth leading cause of cancer mortality worldwide.^1^ The introduction of new treatments for BC^2^ has resulted in peculiar toxicity profiles, requiring a well-organized network of healthcare professionals to effectively manage patient care; furthermore, improved survival rates shifted the focus to improving patients’ quality of life (QoL).^3^^,^^4^ The longer survival of metastatic BC (mBC) now requires a broader perspective to address all BC potential sequelae or therapy side effects, which may affect long-term QoL. Thus, a multidisciplinary approach assessing patient needs, personal resources, and expectations may be effective in tailoring treatment and achieving improvements in survival and QoL outcomes.^5^

Appropriate methods are needed to capture patient subjectivity. The paradigm of person-centered care^6^ has led to the introduction of new tools for patient engagement in the care pathway: among them, patient-reported outcomes measures (PROMs)^7^^,^^8^ can facilitate doctor–patient communication and supportive care delivery.^9^ In oncology, the evolution from paper to electronic PROMS (ePROMs) has been shown to improve symptom control, physical function, QoL, patient compliance, and survival.^10–13^ Moreover, the comprehensive archive of BC-specific PROMs, “PRO4All” has been developed to promote a patient-centered approach in clinical practice, optimizing treatment evaluation and care delivery.^14^ However, standardized quantitative methods^15^ may overlook the dynamism of patient subjective experience during treatments, as they focus on functional scales, symptoms, side effects, and emotional functioning at specific timepoints. In contrast, qualitative approaches, such as patient narratives, dynamically address individual needs and emotions in response to disease and therapies,^16^ thus providing a more comprehensive perspective on the treatment journey.

Narrative medicine (NM)^17^ aims to bridge the gap between biomedical perspective and patients’ subjective illness experiences,^18–21^ thereby promoting a person-centered approach. Since its conceptualization, patient narratives have been increasingly introduced in different areas of medicine, both locally and worldwide.^22^^,^^23^

Advances in digital technologies facilitated a wider integration of NM into clinical practice.^24^^,^^25^ The Digital Narrative Medicine laboratory platform (DNMlab) was launched in Italy in 2016 and used in pilot studies conducted at the Regina Elena National Cancer Institute (Rome, Italy), including patients with cancer and their treating team throughout the care pathway. These studies showed positive results in terms of feasibility and usefulness^26–28^ and paved the way for a more tailored approach to QoL measurement combining physical symptoms with emotional distress, impact on daily life, personal resources, and expectations. Furthermore, a person-based care (PbC) digital pathway has been developed to integrate quantitative ePROMs with qualitative narrative data through the DNMLab platform, allowing to personalize the care pathway according to patients’ experiences and needs.^5^

The “adoption of a digital PbC model for improving QoL of patients with advanced breast cancer” study (PERGIQUAL, clinicaltrials.gov ID: NCT05893368) primarily aimed to evaluate the feasibility and usefulness of the integration of NM with e-PROMs in the PbC digital pathway for patients with HER2-mBC; the secondary aim of the study was to assess the impact on QoL of the PbC digital pathway as perceived and self-reported by patients and treating oncologists.

## Methods

### Study design and patient population

This observational, longitudinal, prospective study was conducted at the University Hospital A. Gemelli IRCCS (Rome, Italy) from May 2023 to February 2024. The study enrolled patients with luminal or triple negative mBC subtypes (estrogen and or progesterone positive or negative and HER2−, immunohistochemical score 0, 1+, or 2+ with nonamplified FISH/SISH], receiving first- or second-line treatment according to national and international guidelines.^29^^,^^30^

Eligible patients were aged ≥18 years, starting first- or second-line treatment for mBC and enrolled before treatment initiation. Additional criteria included a life expectancy ≥24 weeks, knowledge of the Italian language, basic digital literacy, and the ability to understand and sign the informed consent form (ICF). In line with previous studies, basic digital literacy (ability to navigate the web and use e-mail) was considered sufficient to use the platform; patients also had access to dedicated technical assistance by e-mail or telephone. Exclusion criteria included the inability to participate due to psychiatric disorders or severe cognitive deficits in the absence of a caregiver, lack of an email account, or unwillingness or inability to use web-based tools or sign the ICF. For each patient, all relevant data and information were obtained through a comprehensive medical history conducted at the outset of the study and before the initiation of the new treatment (including demographic characteristics, educational background, and clinical history).

### Ethical considerations

The study was performed in accordance with the Declaration of Helsinki. Patients provided written informed consent for study participation, use of the digital platform, and confidential data handling, in compliance with local and European regulations.^31^^,^^32^ Patient priorities, experiences, and preferences were constantly considered throughout the study. Although not involved in study design, patients were informed of findings through direct communication with treating oncologists. The study was approved by the Ethical Committee of the “Agostino Gemelli” University Hospital IRCCS in April 2023 (protocol study n. 5640).

### Intervention

A 2-session training led by NM experts from the multidisciplinary study team familiarized treating oncologists with the digital platform to ensure its effective use, for a total of ≈80 minutes overall. The study team included 8 oncologists with expertise in BC, while the NM team consisted of 2 anthropologists, a laboratory facilitator, a specifically trained epidemiologist–oncologist, 2 doctor–patient communication specialists, and a statistician.

All eligible, consecutively referred patients were invited. Oncologists enrolled patients to access the PbC pathway via the DNMLab digital platform over 8 months (Figure S1). Patients were invited to share their experiences in a digital diary consisting of narrative prompts (Table S1) and complete the EORTC QLQ-C30/+Br23 questionnaire at enrollment and before each treatment cycle; patients could supplement questionnaire responses with free narratives. Patients were considered evaluable for the study if they responded to at least 1 narrative prompt.

Oncologists reviewed and integrated narratives and patient-reported outcomes (PROs) with clinical data, providing feedback and adjusting the care pathway as needed at each visit. At the end of the study, patients and oncologists completed a self-administered questionnaire assessing the feasibility, usefulness, and perceived impact on patient QoL of the PbC pathway. Nonparticipating patients and oncologists were asked to complete a questionnaire detailing their reasons for declining involvement.

### Study endpoints

The feasibility and usefulness of integrating NM and e-PROMs into the PbC pathway constituted the primary endpoint and were evaluated based on patient and oncologist responses to final evaluation questionnaires. Questionnaires were composed of closed questions targeting specific items, evaluated through a Likert scale (1-5)^33^ and open-ended questions soliciting comments.

Moreover, feasibility was also assessed through study compliance, and usefulness through a thematic analysis examining concordance, discordance, and integration between the items emerging from patient narratives and those reported in the ePROs.

The perceived impact of the PbC pathway on QoL was the secondary endpoint and was evaluated based on patient and oncologist feedback to final evaluation questionnaires asking whether the pathway: (1) improved patient QoL, (2) had the potential to bring improvements in QoL, or (3) resulted in no advantage. Furthermore, patients were asked to self-assess their general health status on a 1-7 scale and rate their condition as improving, stable, or worsening.

### Data analysis

Continuous variables were presented as mean ± standard deviation (SD) or median and range, while categorical variables were reported as frequencies and percentages. Thematic analysis of narratives followed the template analysis framework.^34^ Excel matrices were used for categorization and comparison.

The study team and oncologists performed an indepth reading^35^ of patient narratives to provide appropriate responses to patients during their care journey. Narratives were subsequently segmented into meaning units associated with each specific item reported. These units were automatically classified by date and manually analyzed to identify treatment setting and to determine: (1) type, whether they indicated a problem, a resource, or a request to the care team; (2) theme, categorizing content into areas such as physical status, disease progression, psycho-emotional status, and other relevant categories; and (3) description, synthesizing each item in a summary using the patient’s own words.

The team engaged in continuous discussions throughout the analysis with regular peer debriefings. The EORTC QLQ-C30+Br23 questionnaire was analyzed according to the official scoring manual. Each PRO (EORTC QLQ-C30/+Br23) was matched with narratives shared during the same reference period (the 7 days preceding and including the day of questionnaire completion). Correspondence between narratives and PROs was assessed using the following criteria: (1) narrative integration, when narratives included elements not reported in PROs; (2) questionnaire integration, when PROs included content absent in narratives; (3) concordance, when narratives and PROs presented the same elements; and (4) discordance, when narratives and PROs contained contradictory elements. Specifically, concordance occurred when patients described an issue in their diary (eg, needing rest) and selected the same aspect in the questionnaire, while discordance emerged when narratives contradicted questionnaire responses (eg, reporting constipation in narratives but selecting diarrhea in the questionnaire).

The comparison was focused on the presence or absence of specific items rather than their intensity, as the questionnaire self-assessments were rated by patients on a scale, while narratives would have required interpretation by researchers. However, the questionnaire rating scale was still used to enhance the comparison, providing additional insights for deeper analysis.

The study team collectively discussed the results to ensure a thorough interpretation and followed the Standard for Quality Improvement Reporting Excellence (SQUIRE) guidelines.^36^

### Data collection setting and reliability

To strengthen the reliability of both qualitative and quantitative findings, we standardized the setting of data collection through the DNMLab protected digital environment and collegial procedures for narrative analysis.

Narratives prompts, and ePROMs were both self-administered in the same protected digital environment, shared with the care team, ensuring standardized collection without interviewer mediation or contextual influence. Final evaluation questionnaires were delivered separately and completed anonymously, reducing potential reporting bias. Coding of narratives was verified by the study team during the initial phase, and doubtful cases were discussed collegially until consensus was reached, strengthening the reliability of the qualitative analysis.

## Results

A total of 40 consecutive mBC patients were invited to participate in the study; 2 refused and 38 accepted and were enrolled. Twenty-nine (76.3%) patients actively participated by responding to at least 1 narrative prompt. Table 1 summarizes the baseline sociodemographic and clinical data of the participants.

**Table 1.** Baseline sociodemographic and clinical data of patients.

	Patients (*n* = 29)
**Age, years**	57 (49-64)
**BMI, kg/m^2^**	22.9 (20.3-25.7)
**Menopausal status (%)**	
** Premenopausal**	8 (27.6)
** Postmenopausal**	21 (72.4)
**Level of education (%)**	
** Middle school diploma**	1 (3.4)
** High school diploma**	13 (44.8)
** University degree**	12 (41.4)
** (missing)**	3 (10.3)
**Parity (%)**	
** 0**	1 (3.4)
** 1**	8 (27.6)
** 2**	12 (41.4)
** 3**	0
** 4**	1 (3.4)
** (missing)**	7 (24.1)
**Familiarity (%)**	
** No**	11 (37.9)
** Yes**	18 (62.1)
**Previous nonbreast malignancies (%)**	
** No**	25 (86.2)
** Yes**	3 (10.3)
** (missing)**	1 (3.4)
**Previous malignancies (%)**	
** No**	22 (75.9)
** Yes**	7 (24.1)
**Time from diagnosis, years**	2 (0-4)
**Smoker (%)**	
** No**	23 (79.3)
** Yes**	5 (17.2)
** (missing)**	1 (3.4)
**Oral contraceptive user (%)**	
** No**	17 (58.6)
** Yes**	11 (37.9)
** (missing)**	1 (3.4)
**Breast cancer subtype (%)**	
** HR+/HER2-**	21 (72.4)
** HR−/HER2-**	8 (27.6)
**Type of treatment**	
** HR+/HER2− (%)**	
**CDK4/6 inhibitors + hormonal treatments (first line)**	17 (58.6)
** Fulvestrant monotherapy (second line)**	2 (6.9)
** Capecitabine (second line)**	1 (3.4)
** Elacestrant (second line)**	1 (3.4)
** HR−/HER2− (%)**	
** Chemotherapy ± pembrolizumab (first line)**	6 (75)
** Chemotherapy ± atezolizumab (first line)**	1 (12.5)
** Capecitabine (second line)**	1 (12.5)

Data are presented as *n* (%) or median (IQR).

Abbreviations: BMI, body mass index; IQR, interquartile range.

The median age was 57 years (49-67), with 72.4% being postmenopausal. Most patients had completed either high school (44.8%) or university (41.4%) education, were nonsmokers (79.3%), had predominantly 2 children (41.4%) or 1 child (27.6%), had no history of previous malignancies (75.9%), but did have a family history of cancer (62.1%). At the end of the study, 15 patients (52%) were still on active treatment without evidence of disease progression.

Eight treating oncologists were invited to participate in the study, with 6 actively taking part. Two declined due to the need for thorough training and adequate organizational support.

### Compliance and feasibility assessment of the PbC pathway

The incidence of responses to narrative prompts was 74% (377 responses out of 509 prompts sent), while 79 narrative texts were shared in the absence of any prompts.

Twenty-five out of 29 patients completed the initial PRO questionnaire. Sixteen, fifteen, and sixteen reported QoL-related data during the first treatment cycle, the second treatment cycle, and at the end of the study, respectively. Twenty-one patients (72.4%) completed the final evaluation questionnaire; 5 did not complete it due to clinical deterioration, and 3 due to poor compliance with digital technology. All participating oncologists completed the final evaluation questionnaire.

The overall feasibility assessment of the PbC digital pathway was favorable for both patients and oncologists (Table 2).

**Table 2.** Patients’ and treating oncologists’ feasibility assessment of the digital pathway.

		Mean score ± SD
**Patients (*N* = 21)**	Diary ease of use	4.6 ± 0.50
	Easy comprehension of diary tools	4.5 ± 0.75
	Adequate pre-existing skills for diary use	4.5 ± 0.51
	Possibility to share important information	4.6 ± 0.75
	Communication of personal elements that couldn’t/wouldn’t be shared verbally	3.9 ± 1.14
**Oncologists (*N* = 6)**	Diary ease of use	4.7 ± 0.52
	Diary immediacy and comprehensibility	4.3 ± 0.82
	Time management to propose/evaluate the diary during clinical examinations	4.5 ± 0.55
	Optimized clinical examination (length)	4.7 ± 0.52
	Optimized clinical examination (quality)	4.3 ± 0.52

Abbreviations: PbC, person-based care, SD, standard deviation.

Patients positively rated the ease of use of the digital diary (4.6 ± 0.50) and its effectiveness in conveying important information to the care team that they might not have been able to communicate verbally (4.6 ± 0.75). Patients gave less consistent ratings to the diary’s ability to help them express content they felt they could not communicate verbally (3.9 ± 1.14). Oncologists positively rated the diary for ease of use (4.7 ± 0.52), considering its compatibility with visit time management (4.5 ± 0.55). They also positively rated it for optimizing clinical examination in terms of both length (4.7 ± 0.52) and quality of care (4.3 ± 0.52).

### Usefulness assessment of the PbC digital pathway

#### Patients’ and oncologists’ evaluations

In final evaluation questionnaires, the usefulness assessment was favorable (±4.2) for most indicators (Table 3). Patients considered the integration of narratives and e-PROMs as useful to better convey disease- and treatment-related experience (4.2 ± 0.94). There was stronger agreement on narrative as a mean to express experiences not captured by the questionnaire (4.5 ± 0.68) and the recommendation to include the digital diary in clinical practice (4.6 ± 0.92). The digital diary’s effectiveness in improving patients’ ability to cope with the situation showed less consistent results (3.8 ± 1.14).

**Table 3.** Patients’ and treating oncologists’ usefulness assessment of the PbC digital pathway.

		Mean score ± SD
**Patients (*N* = 21)**	Opportunity to express one’s point of view	4.2 ± 1.00
	Perception of more effective care management by the healthcare team	4.2 ± 1.12
	Perception of improved understanding of personal needs by the care team	4.3 ± 0.97
	Enhanced awareness of one’s health condition and needs	4.1 ± 0.96
	Improved ability to cope with the situation	3.8 ± 1.14
	Recommendation to include the digital diary in clinical practice	4.6 ± 0.92
	Usefulness of both free narratives and questionnaires for deepening understanding of illness and treatment experiences	4.2 ± 0.94
	Opportunity to share experiences not covered by the questionnaire	4.5 ± 0.68
	Improved elaboration of responses to the questionnaire	4.3 ± 0.78
	Improved reflection on and dealing with the critical aspects reported in the questionnaire	4.1 ± 1.04
**Oncologists (*N* = 6)**	Improved communication with the patient	4.8 ± 0.41
	Improved care relationship	4.5 ± 1.22
	Deeper knowledge of the patient	5.00 ± 0.00
	Improved therapeutic alliance	4.3 ± 0.82
	Focus on key points of the care pathway	4.2 ± 0.75
	Improved care outcomes	4.2 ± 0.41
	Improved team relationship	3.7 ± 0.82
	Usefulness of both free narratives and questionnaires for deepening understanding of illness and treatment impact	4.3 ± 0.52
	Opportunity to identify aspects not typically detected by questionnaires	5.00 ± 0.00
	Improved understanding of the impact and duration of critical issues through narrative tools	4.3 ± 0.82
	Opportunity to identify discordant aspects of questionnaire responses	3.5 ± 1.05

Abbreviations: PbC, person-based care, SD, standard deviation.

Oncologists identified a deeper understanding of the patient (5.0 ± 0.00), the opportunity to gather details typically not captured by PROMs (5.0 ± 0.00), and improved patient communication (4.8 ± 0.41) as the main advantages of integrating narratives and quantitative tools. They also highlighted that the PbC pathway enabled a more comprehensive exploration of disease- and treatment-related effects (4.3 ± 0.52). In several cases, narratives revealed needs and concerns that had not emerged during routine visits, making it possible for clinicians to consider them and provide timely responses, such as referral to psycho-oncology, adjustment of supportive care, or reorganization of follow-up visits. This complementarity with ePROMs supported more personalized care and strengthened the patient–clinician relationship. The mean score was slightly lower (3.7 ± 0.82) for the enhancement of team relationships.

#### Comparative analysis of patient narratives and PROs

A database of 1660 items was generated from patient narratives (Table 4). Items identified as “resources” were most common (52%), followed by “problems” (42%), while “requests” to the care team were rare (6%).

**Table 4.** Thematic analysis of patient narratives.

		Typology			% on total items
		Problem (%)	Request	Resource	
**Themes**	Disease course	27 (50)	7 (13)	20 (37)	54 (3.3)
	Treatments and diagnostic exams	70 (31)	57 (25)	97 (43)	224 (13.5)
	Care pathway and treating oncologists	16 (15)	17 (16)	73 (79)	106 (6.4)
	Physical status	293 (79)	4 (1)	76 (20)	373 (22.5)
	Psycho-emotional status	176 (44)	5 (1)	221 (55)	402 (24.2)
	Sexuality	1 (50)	1 (50)	-	2 (0.1)
	Lifestyle	14 (31)	5 (11)	26 (58)	45 (2.7)
	Activities	19 (12)	3 (2)	131 (86)	153 (9.2)
	Values and believes	1 (3)	-	35 (97)	36 (2.2)
	Expectations and projects	12 (16)	2 (3)	59 (81)	73 (4.4)
	Social environment	13 (87)	1 (7)	1 (7)	15 (0.9)
	Relationships and affections	44 (25)	2 (1)	129 (74)	175 (10.5)
	Economic coverage	1 (50)	-	1 (50)	2 (0.1)
**% on total items**		687 (42)	104 (6)	869 (52)	1.660 (100)

Results are expressed as *N* (%).

Narratives clustered around 5 main themes: (1) psycho-emotional aspects (24% of total narratives), with resources (55%) exceeding problems (44%); (2) physical condition (22%), consisting mostly of problems (79%); (3) treatment and diagnostic tests (13%), distributed among resources (43%), problems (31%), and requests (25%); (4) relationships and affection (11%), largely represented by resources (74%); and (5) daily activities (9%), primarily composed of resources (86%).

Conversely, references to sexual and economic spheres were almost absent.

The overall QoL evaluation revealed that, despite a slight decline from the baseline to the first assessment (mean score 65.3 and 59.4, respectively), there was an improvement in the overall scoring by the end of the study (final score 69.8). Besides role functioning, most of the domains (physical, emotional, cognitive, and social functioning) showed an improvement at the final assessment. However, the response to symptoms varied: nausea, pain, insomnia, and appetite loss improved, while fatigue, dyspnea, constipation, and diarrhea worsened over time. In the Br23 questionnaire, patients noted improvements in arm and breast symptoms, body image, and future perspective. Systemic therapy side effects remained stable over time, while there was a negative impact on sexual functioning and enjoyment. A complete report of the QoL trend is available in Table S2.

The narratives and questionnaires were also analyzed for concordance and discordance (Table 5). For this comparison, only narratives shared within 1 week before questionnaire completion were considered.

**Table 5.** Narrative and questionnaire analysis for concordance, discordance, and integration.

	Typology			% on total items
	Problem	Request	Resource	
**Concordance**	81 (5.6)	-	10 (2.2)	91 (4.8)
**Discordance**	8 (0.6)	-	2 (0.4)	10 (0.5)
**Narrative integration**	225 (15.5)	2 (100)	353 (79)	580 (30.5)
**E-proms integration**	1137 (78.3)	-	82 (18.3)	1219 (64.2)
**% on total items**	1451 (76.4)	2 (0.1)	447 (23.5)	1900 (100)

Results are expressed as *N* (%).

Ninety-one instances of concordance (4.8%) and 10 instances of discordance (0.5%) were identified between narratives and questionnaires. In 580 cases (30.5%), narratives provided additional information not captured by questionnaires, particularly regarding resources and personal experiences. Conversely, in 1219 cases (64.2%), questionnaires supplemented narratives, often when patients didn’t mention in their diaries symptoms they marked in questionnaires. Questionnaires played a more prominent integrative role (78.4%) compared to narratives (15.5%) for “problems,” while for “resources,” narratives had a greater integrative role (79%) than questionnaires (18.3%). Notably, 67% of the “problem” items identified only in questionnaires were rated as merely “a little” critical by patients, which may explain why these issues weren’t spontaneously mentioned in their narratives, while only 26% were rated as “quite a bit” and 7% as “very much” on the rating scale.

#### Perceived impact on patient QoL

Among the 21 patients who completed the final evaluation questionnaire, 14 self-assessed their general health status with a median score of 5 (2-6), with the disease rated as “stable” or “improving.” Ten out of 21 patients perceived that the PbC pathway contributed to an improvement in their QoL (Table 6). Five out of 21 patients reported no perceived improvement in their QoL: 4/5 found the PbC pathway inadequate, while 1/5 reported a worsening in clinical condition.

**Table 6.** Patients’ and treating oncologists’ assessment on the perceived impact on QoL of the PbC digital pathway.

	All patients (*N* = 21)
**Improvement in perceived QoL**	10 (48)
** Opportunity to better understand patient needs (4/10)**	
***Writing in the diary allowed me not to feel alone and to be understood as the treatment continued.***	
** Improvement in self-care (3/10)**	
***It was a wonderful experience. It was a stimulating experience, and I will miss it. It made me feel more confident about my path and, at the same time, much more careful and responsible in following and dealing with treatment.***	
** Opportunity to better manage disease and treatment (3/10)**	
***The diary really helps one to reflect on the situation, so it is a very valuable tool. The caring relationship, of course, is greater through the diary, as one can bring up difficulties as they arise without waiting to be examined, and, what’s more, it is an almost immediate strategy through which the medical team grasps the patient’s needs to discuss them at the monthly meeting.***	
**Contribution to future improvement**	6 (28)
** Future possibility for the care team to better understand the patient (5/6)**	
***I did not use this opportunity enough; I responded to the stimuli but did not, for example, use the possibility of entering the stimuli myself. The doctor read my answers, even though we only shared impressions once, probably because I already have many things to ask during the visit. However, I think it would be useful to continue; perhaps I need more time to better understand how to use this tool.***	
** Future possibility for both patient and care team to better understand the disease and treatment (1/6)**	
***The diary allowed me to face the psychological side of the illness… I realized that there are very fragile parts of myself that I had not yet focused on! And I realized that I needed psychological support, but not so much for everyday life, but to be able to cope with the dark moments I will ‘inevitably’ come up against.***	
**No change in perceived QoL**	5 (24)
** Inappropriate tool to communicate patient needs (4/5)**	
***It is a very impersonal tool, lacking a genuine connection with the care team. The prompt questions are often vague and, at times, increased my anxiety about the future.***	
** Disease exacerbated by treatments^a^ (1/5)**	

	All oncologists (N = 6)
**Improvement in patient perceived QoL**	4 (67)
** Opportunity to better understand patient needs (2/4)**	
***[…] In some cases, it allowed us to focus on situations that emerged from the narrative diaries, and more than one patient expressed the wish to be able to continue to be involved in projects of this type because they helped her to feel heard.***	
** Opportunity to better understand patient needs (2/4)**	
***In most patients, it succeeds in delineating well the impact of the patient’s diagnosis and treatment with the advantage of better identifying any ‘critical’ moments […].***	
**Contribution to future improvement**	2 (33)
** Future opportunity for the patient to better understand her needs (1/2)**	
***If the team is involved, the tool works better.***	
** Future opportunity to tailor the care pathway (1/2)**	
***I believe that this tool can certainly help clinicians in many ways (personalization of the treatment and care pathway, greater attention and listening to patients’ needs, organization of visits, better knowledge of “people” and not just patients). Not for all patients it may be useful, for some it has caused more anxiety to have to think frequently about their current state or to answer some more personal questions.***	

Data are expressed as *N* (%). Abbreviations: PbC, person-based care, QoL, quality of life.

a No free narrative text available.

Oncologists reported that the PbC pathway had either led to an improvement in patient QoL (4/6) or could potentially do so in the future (2/6), as it allowed both patients and clinicians to gain deeper insights and personalize the care pathway.

## Discussion

The PERGIQUAL study introduced an innovative approach that integrates quantitative ePROMs with qualitative narrative methods to dynamically capture the various aspects of the patient’s experience throughout the care pathway.

Activity on the digital platform was high, with a substantial number of narrative prompts sent and a response rate exceeding 70%. The 79 free-text narratives underscored patients’ appreciation for adopting narrative practices in their care pathway.

The primary endpoint of the study was to assess the feasibility and usefulness of integrating NM and e-PROMs into a single digital patient-listening pathway (PbC pathway). This endpoint was validated in previous studies focused on the digital narrative diary^26–28^ and confirmed in this study for the PbC pathway. Both patients and oncologists found it easy to use and effective in improving communication. Patients found the digital diary accessible and appreciated its effectiveness in conveying important information to the care team; nonetheless, some patients highlighted concerns about the lack of interaction with treating oncologists and discomfort with self-reflection.

Oncologists, in turn, reported that the PbC pathway allowed them to gather previously unaddressed information, facilitating more informed and personalized care decisions. However, they noted challenges in integrating this information within the care team. While the PbC pathway effectively revealed important patient-specific insights, integrating it into team-based care was less straightforward, as it relies on the individual willingness of healthcare professionals to incorporate and apply this information in daily practice. This difficulty may also reflect reasons given by the 2 oncologists who declined participation in the study, that is, the need for thorough training and adequate organizational support to fully leverage the tool’s potential.

Integrating narratives and e-PROMs within the same digital pathway offered a unique opportunity for both patients and clinicians to address the physical, emotional, and social aspects of the disease. This facilitated a more dynamic and personalized care approach. Analysis of patient narratives revealed that while e-PROMs effectively captured the physical aspects of the disease, the narrative diary allowed patients to express psycho-emotional and personal experiences not typically captured by questionnaires. This complementary approach enriches the overall understanding of patient needs, enhancing the care process. Indeed, narratives crucially contributed to uncovering coping resources and psycho-emotional aspects that e-PROMs alone did not address.

A few specific requests addressed to the care team emerged from patient narratives. This may be because the digital diary was primarily used as a monitoring tool rather than for interaction. Patient narratives allowed for a dynamic capture of various aspects of their perceptions of “oncological life” during treatments, which may differ at different moments in the daily patient experience, both regarding treatment side effects and disease-specific concerns. Moreover, narratives highlighted side effects that patients might otherwise forget or downplay during clinical visits.

The questionnaire helped patients focus on issues—particularly those related to physical health—that might not have emerged in narratives. However, 67% of the “problem” items identified exclusively in the questionnaires were rated as “a little” critical, suggesting they are important but not top priorities. Nonetheless, recognizing these issues can help the care team prevent the progression of signs and symptoms. It is worth highlighting that patients do not spontaneously discuss sexuality and financial toxicity in their narratives. To gather information on these crucial topics, they must be explicitly included in prompts, allowing for insights that extend beyond what standard questionnaires capture.

The comparison between PROs and narratives enhanced the value of integrating both qualitative and quantitative methods for active listening, personalizing care pathways, and managing toxicities. Sole reliance on the narrative diary may overlook emerging issues that could become significant over time. Conversely, e-PROMs alone are inadequate, as they fail to capture subjective resource-related items crucial for patients. Furthermore, e-PROMs fail to capture symptom duration accurately due to their intermittent assessment schedule. Research has shown that the repeated use of quantitative assessment tools can result in patient fatigue and decreased compliance.^37^ In contrast, narrative monitoring creates a supportive environment where patients can express themselves freely in their own terms, encouraging more authentic participation and providing deeper insights into their lived experience.

It is noteworthy that e-PROMs analysis was not designed to provide statistical comparisons between different timepoints, due to small sample size and variable number of answers for each item/domain, but rather to offer insights into the overall trend in QoL, both general and breast-specific.

As for the secondary study endpoint, 48% of patients reported a perceived improvement in QoL. Among them, 30% attributed this improvement to the care team’s enhanced understanding of their needs, 40% to better self-management, and 30% to a combined effect, with both patients and clinicians gaining a clearer understanding of how to address their situation. This variety highlights the capability of such tools to meet different patient needs and stresses the importance of personalized care. Twenty-eight percent of patients noted no improvement but believed the tool could enhance future care. Similarly, 4 out of 6 oncologists reported that the PbC pathway positively impacted patient QoL, helping both patients and clinicians gain a clearer understanding of the care pathway and tailor interventions accordingly. The study successfully met its aims, particularly in personalizing the care pathway to address patients’ needs.

Despite these positive findings, 24% of patients reported no perceived impact on their QoL, as they felt that the digital diary was not the right tool for this purpose. In line with the findings of a previous survey,^38^ 1 patient described it as too impersonal and noted that the prompts caused her anxiety about the future. This suggests that while the PbC pathway offers considerable advantages, it should not be the sole method of patient engagement, as it may not suit all patients. Future adaptations should include clearer communication about the tool’s objectives at the time of invitation, timely feedback on narratives during visits, alternative modalities beyond writing, and refinement of prompts based on patient input.

Its application may require adjustments to better align with patient characteristics, illness phase, type of therapy, disease severity, and hospital organizational factors, such as the number of dedicated oncologists and day hospital arrangements. As highlighted by a qualitative study,^39^ the acceptance of innovative digital tools by patients and healthcare professionals requires time and support in understanding their feelings of insecurity.

The strength of the study is that, by integrating NM approach with e-PROMs, the PbC pathway enabled both patients and oncologists to engage more deeply with the mBC illness experience, unveiling coping resources and psycho-emotional aspects and fostering a more tailored and empathetic care.

Nonetheless, this study has some limitations. The small number of participants, both patients and oncologists, and the single-center design restrict the generalizability of the findings, which may partly reflect the organizational setting of our institution. Although the requirement for basic digital literacy may have introduced a selection bias, the platform itself proved easy to use for both patients and clinicians. Broader applicability therefore, depends less on the technology than on organizational resources, with ≈80 minutes of training required for clinicians to use the platform, ≈15 minutes needed on average to review each patient's answer prompt and PRO response, and the suitability of the written format for all patients. The advanced disease status may also explain the limited number of patients enrolled, together with possible hesitation to share personal experiences through narrative. Finally, the PbC pathway was not tailored to patient characteristics, which may account for its limited perceived impact on QoL for some participants, and oncologists emphasized the need for dedicated training and organizational support to fully integrate NM into practice. Based on the results of this study, the enhancement of the PbC pathway within multidisciplinary teams was identified to more effectively address individual patient needs. In response, the In Person study (NCT05893368), conducted at the A. Gemelli University Hospital IRCCS involved integrated therapy teams and introduced narrative diaries designed to track movement, nutrition, and sleep patterns. An additional, initially unplanned, outcome of this study was the development of a narrative analysis framework that systematically identifies and categorizes narrative elements such as Resources or Problems. Drawing the approach used in the PERGIQUAL study, this methodology organizes narrative elements into thematic domains (eg, disease course, physical status, psycho-emotional status, and others as detailed in Table 4) while preserving patients’ linguistic choices, including emotional expressions and metaphors. This framework was subsequently used to train a generative artificial intelligence system for real-time narrative analysis to improve the sharing of narrative data within the care team and their integration with clinical data for personalized treatment. This capability has recently been implemented in clinical practice at A. Gemelli University Hospital IRCCS through the Narr-Arti PbC Pathway.

## Conclusions

In this study, integrating NM and ePROMs in the PbC pathway has been shown as feasible and useful in the clinical management of patients with HER2(-) mBC. The PbC pathway demonstrated its effectiveness in the perceived impact on QoL for a considerable portion of patients with mBC, while fostering a more personalized and empathetic care. This pilot experience suggests that, with adequate clinician training, a digital PbC model combining narratives and ePROMs may improve patient assessment and supportive care in metastatic breast cancer; however, its generalizability remains limited, and confirmation in larger multicenter studies is required. Furthermore, the findings suggest that integrating narratives and PROs offers a comprehensive view of patient care, but refinement is needed to identify where this tool is most beneficial and to involve different healthcare professionals to tailor patient treatment and care.

## Supplementary Material

oyaf367_Supplementary_Data

## Data Availability

Data supporting the findings of this study are available from the corresponding author upon reasonable request.
