# Supplementary material for: Digital integration of narrative medicine and patient-reported outcome measures to improve understanding of quality of life in metastatic breast cancer: the PERGIQUAL study
Source: Oncologist. 2025 Nov 5;30(12):oyaf367. doi: 10.1093/oncolo/oyaf367 (PMC12687594; doi:10.1093/oncolo/oyaf367)
Supplement: oyaf367_Supplementary_Data [file oyaf367_supplementary_data.docx]

**Supplementary file 1** – Study design of the PERGIQUAL study


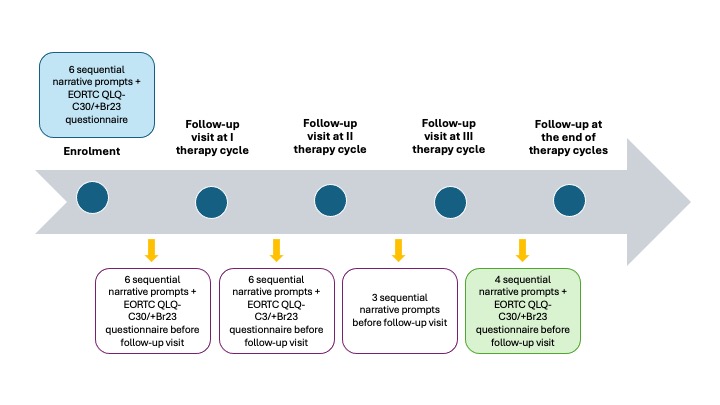


**Supplementary file 2** – Narrative prompts used in the digital narrative diary

| **Timeframe** | **Purpose** | **Narrative prompts** | **English translation of narrative prompts** |
| --- | --- | --- | --- |
| **Baseline prompt list** | Six sequential narrative prompts to focus on patient’s illness experience | L’esperienza della malattia è diversa per ognuno. Qui vi racconto la mia. | The illness experience is different for everyone. Here, I tell you about mine. |
|  |  | Le cure: come le ho vissute in passato e cosa mi aspetto ora. | Treatments: how I experienced them in the past and what I expect now. |
|  |  | La mia vita quotidiana e i miei impegni nei prossimi mesi. | My daily life and my tasks in the coming months. |
|  |  | Mi aiuta… Non mi aiuta… | It helps me… It doesn’t help me… |
|  |  | Grazie per aver condiviso la Sua esperienza. Le chiediamo ora di assegnare un punteggio ad alcuni aspetti specifici, rispondendo a un questionario che trova qui*. Se lo ritiene utile, può commentare e integrare le risposte che darà con un testo libero, rispondendo a questo messaggio. Se dopo aver risposto al questionario, non avrà nulla da aggiungere, usi il tasto “Ignora” per continuare a usare il diario narrativo. | Thank you for sharing your experience. We now ask you to rate some specific aspects by answering a questionnaire that you can find here*. If you find it helpful, you can comment and supplement your answers with a free text by replying to this message. If you have nothing more to add after answering the questionnaire, use the ‘Ignore’ button to continue using the narrative diary. |
|  |  | Al prossimo incontro vorrei sapere… | At the next visit, I would like to know… |
| **Prompt list at follow-up visit at I therapy cycle** | Six sequential narrative prompts with different timeframes to focus on therapy impact and changes in patient’s daily life | 1. *At therapy start* 2. Ho iniziato la nuova terapia e… | I started the new therapy and… |
|  |  | 1. *After a week* 2. In questa settimana… | 1. This week… |
|  |  | 1. *Two days after* 2. La mia vita quotidiana… | 1. My daily life… |
|  |  | *After one week*  Per me è importante… | 1. For me, it is important… |
|  |  | 1. Grazie per aver condiviso la sua esperienza in queste prime settimane di terapia. Le chiediamo ora di rispondere nuovamente al questionario che già conosce, per capire se è cambiato qualcosa. Lo trova qui*. Se lo ritiene utile, può commentare e integrare le risposte che darà con un testo libero. Se dopo aver risposto al questionario, non avrà nulla da aggiungere, usi come sempre il tasto “Ignora” per continuare a usare il diario narrativo. | 1. Thank you for sharing your experience during these first weeks of therapy. We now ask you to answer the questionnaire you are already familiar with, to see if anything has changed. You can find it here*. If you find it helpful, you can comment and supplement your answers with a free text. If you have nothing more to add after answering the questionnaire, use the ‘Ignore’ button as usual to continue using the narrative diary. |
|  |  | 1. Al prossimo incontro vorrei sapere… | 1. At the next visit, I would like to know… |
| **Prompt list at follow-up visit at II therapy cycle** | Six sequential narrative prompts with different timeframes to focus on therapy impact and changes in patient’s daily life | 1. In questo periodo mi sento… | 1. During this period, I feel… |
|  |  | 1. *After a week* 2. Ora penso che le cure… | Now I think that the treatments… |
|  |  | 1. *After two days* 2. In questo momento mi aiuta… Non mi aiuta… | 1. At this moment, it helps me… It doesn’t help me… |
|  |  | 1. *After four days* 2. Pensando ai prossimi mesi… | Thinking about the coming months… |
|  |  | 1. Grazie. Come nelle scorse settimane, le chiediamo ora di rispondere nuovamente al questionario. Lo trova qui*. Se lo ritiene utile, può commentare e integrare le risposte che darà con il testo libero. Se dopo aver risposto al questionario, non avrà nulla da aggiungere, usi come sempre il tasto “Ignora” per continuare a usare il diario narrativo. | 1. Thank you. As in the previous weeks, we now ask you to answer the questionnaire again. You can find it here*. If you find it helpful, you can comment and supplement your answers with a free text. If you have nothing more to add after answering the questionnaire, use the ‘Ignore’ button as usual to continue using the narrative diary. |
|  |  | 1. Mi aspetto che al prossimo incontro… | 1. For the next visit, I expect that… |
| **Prompt list at follow-up visit at III therapy cycle** | Three sequential narrative prompts with different timeframes to focus on therapy impact, changes in daily life, and patient’s specific needs | 1. Dopo questi mesi di cure mi sono resa conto che… | After these months of treatments, I realised that… |
|  |  | 1. *After 10 days* 2. Nei prossimi mesi potrei avere bisogno di… | In the coming months, I might need… |
|  |  | 1. Grazie per aver condiviso le sue esigenze. Durante tutto il percorso di cura, può condividere riflessioni, esperienze e nuove esigenze utilizzando il tasto ‘Aggiungi una storia’. Aggiunga i suoi eventuali commenti rispondendo a questo messaggio o lo ignori per continuare a usare il diario. | 1. Thank you for sharing your needs. Throughout the care pathway, you can share reflections, experiences, and new needs by using the ‘Add a story’ button. Add any comments by replying to this message or ignore it to continue using the diary. |
| **Prompt list at follow-up visit at the end of therapy cycles** | Four sequential narrative prompts to focus on therapy impact, changes in daily life, and patient’s specific needs | 1. In questo periodo la mia vita quotidiana… | 1. During this period, my daily life… |
|  |  | 1. Dopo tutti questi mesi posso dire che… | 1. After all these months, I can say that… |
|  |  | 1. Grazie. Come negli scorsi mesi, le chiediamo ora di rispondere nuovamente al questionario. Lo trova qui*. Se lo ritiene utile, può commentare e integrare le risposte che darà con il testo libero. Se dopo aver risposto al questionario, non avrà nulla da aggiungere, usi come sempre il tasto “Ignora” per continuare a usare il diario narrativo. | 1. Thank you. As in previous months, we now ask you to answer the questionnaire again. You can find it here*. If you find it helpful, you can comment and supplement your answers with a free text. If you have nothing more to add after answering the questionnaire, use the ‘Ignore’ button as usual to continue using the narrative diary. |
|  |  | 1. Al prossimo incontro vorrei parlassimo di… | 1. At the next visit, I would like us to talk about… |
| PERGIQUAL, New Model for Integrating Person-based Care (PbC) in the Treatment of Advanced HER2-negative Breast Cancer.  * Link to EORT QLQ-C30/+Br23 questionnaire. | | | |

**Supplementary file 3** – Quality of life trend in PERGIQUAL study: EORTC QLQ-C30/+Br23 scores

**SF3.a** – EORTC QLQ-C30 questionnaire scores

|  |  | **QOL** | **PF** | **RF** | **EF** | **CF** | **SF** | **FA** | **NV** | **PA** | **DY** | **SL** | **AP** | **CO** | **DI** | **FI** |
| --- | --- | --- | --- | --- | --- | --- | --- | --- | --- | --- | --- | --- | --- | --- | --- | --- |
| **Baseline** | N | 25 | 25 | 25 | 25 | 25 | 25 | 25 | 25 | 25 | 25 | 25 | 25 | 25 | 25 | 25 |
|  | Mean | **65.3** | 76.5 | 74.7 | 73.7 | 88.0 | 78.0 | 33.3 | 12.0 | 26.7 | 14.7 | 33.3 | 16.0 | 12.0 | 9.3 | 10.7 |
|  | SD | 24.1 | 21.4 | 28.1 | 18.0 | 16.3 | 28.3 | 28.3 | 19.6 | 31.2 | 21.7 | 30.4 | 29.1 | 21.3 | 22.6 | 23.0 |
| **I therapy cycle** | N | 16 | 16 | 16 | 16 | 16 | 16 | 16 | 16 | 16 | 16 | 16 | 16 | 16 | 16 | 16 |
|  | Mean | **59.4** | 78.8 | 68.8 | 69.3 | 82.3 | 69.8 | 32.6 | 10.4 | 25.0 | 16.7 | 33.3 | 20.8 | 16.7 | 20.8 | 8.3 |
|  | SD | 24.7 | 18.7 | 28.5 | 19.7 | 23.9 | 29.9 | 25.8 | 13.4 | 27.9 | 21.1 | 34.4 | 26.9 | 24.3 | 24.0 | 19.2 |
| **II therapy cycle** | N | 15 | 15 | 15 | 15 | 15 | 15 | 15 | 15 | 15 | 15 | 15 | 15 | 15 | 15 | 15 |
|  | Mean | **63.3** | 76.0 | 76.7 | 67.2 | 85.6 | 74.4 | 43.0 | 15.6 | 35.6 | 15.6 | 26.7 | 17.8 | 15.6 | 17.8 | 11.1 |
|  | SD | 23.3 | 25.3 | 30.1 | 28.6 | 23.5 | 32.0 | 29.1 | 17.2 | 32.7 | 30.5 | 25.8 | 24.8 | 24.8 | 21.3 | 20.6 |
| **End of therapy cycles** | N | 16 | 16 | 16 | 16 | 16 | 16 | 16 | 16 | 16 | 16 | 16 | 16 | 16 | 16 | 16 |
|  | Mean | **69.8** | 79.2 | 72.9 | 83.3 | 89.6 | 80.2 | 36.8 | 8.3 | 18.8 | 20.8 | 27.1 | 8.3 | 18.8 | 10.4 | 2.1 |
|  | SD | 23.9 | 16.5 | 31.5 | 16.4 | 14.8 | 24.5 | 25.9 | 12.2 | 21.8 | 26.9 | 32.7 | 25.8 | 29.7 | 20.1 | 8.3 |
| *AP, appetite loss; CF, cognitive functioning; CO, constipation; DI, diarrhea; DY, dyspnea; EF, emotional functioning; FA, fatigue; FI, financial difficulties; N, number of respondent patients; NV, nausea and vomiting; PA, pain; PF, physical functioning; QoL, quality of life; RF, role functioning; SD, standard deviation; SF, social functioning; SL, insomnia.* | | | | | | | | | | | | | | | | |

**SF3.b** – Br23 questionnaire scores

|  |  | **ST** | **HL** | **AS** | **BS** | **BI** | **FU** | **SEF** | **SEE** |
| --- | --- | --- | --- | --- | --- | --- | --- | --- | --- |
| **Baseline** | N | 25 | 8 | 25 | 25 | 25 | 25 | 25 | 10 |
|  | Mean | 19.7 | 41.7 | 17.3 | 16.3 | 77.7 | 41.3 | 14.0 | 40.0 |
|  | SD | 15.2 | 15.4 | 21.8 | 24.8 | 29.5 | 30.9 | 18.4 | 21.1 |
| **I therapy cycle** | N | 16 | 9 | 16 | 16 | 16 | 16 | 16 | 7 |
|  | Mean | 16.4 | 33.3 | 9.0 | 9.4 | 84.9 | 39.6 | 16.7 | 42.9 |
|  | SD | 10.0 | 37.3 | 15.3 | 14.9 | 17.0 | 30.4 | 20.2 | 31.7 |
| **II therapy cycle** | N | 15 | 7 | 15 | 15 | 15 | 15 | 15 | 7 |
|  | Mean | 23.1 | 52.4 | 17.0 | 13.3 | 75.6 | 40.0 | 18.8 | 52.3 |
|  | SD | 14.6 | 37.8 | 19.6 | 26.1 | 29.1 | 28.7 | 22.2 | 17.8 |
| **End of therapy cycles** | N | 16 | 7 | 16 | 16 | 16 | 16 | 16 | 8 |
|  | Mean | 20.5 | 42.9 | 10.4 | 8.9 | 79.2 | 56.3 | 18.7 | 50.0 |
|  | SD | 12.6 | 37.1 | 17.4 | 10.3 | 22.4 | 23.5 | 20.6 | 30.8 |
| *AS, arm symptoms; BI, body image; BS, breast symptoms; FU, future perspective; HL, upset by hair loss; N, number of respondent patients; SD, standard deviation; SEE, sexual enjoyment; SEF, sexual functioning; ST, systemic therapy side effects.* | | | | | | | | | |
